# The Impact of Sub-Millisecond Damage Fixation Kinetics on the In Vitro Sparing Effect at Ultra-High Dose Rate in UNIVERSE

**DOI:** 10.3390/ijms23062954

**Published:** 2022-03-09

**Authors:** Hans Liew, Stewart Mein, Thomas Tessonnier, Amir Abdollahi, Jürgen Debus, Ivana Dokic, Andrea Mairani

**Affiliations:** 1 Clinical Cooperation Unit Radiation Oncology, German Cancer Research Center (DKFZ), 69120 Heidelberg, Germany; h.liew@dkfz-heidelberg.de (H.L.); juergen.debus@med.uni-heidelberg.de (J.D.); 2 Division of Molecular and Translational Radiation Oncology, National Center for Tumor Diseases (NCT), Heidelberg University Hospital, 69120 Heidelberg, Germany; s.mein@dkfz-heidelberg.de (S.M.); a.amir@dkfz-heidelberg.de (A.A.); i.dokic@dkfz-heidelberg.de (I.D.); 3 Heidelberg Institute of Radiation Oncology (HIRO), German Cancer Research Center (DKFZ), 69120 Heidelberg, Germany; 4 German Cancer Consortium (DKTK), 69120 Heidelberg, Germany; 5 Heidelberg Ion-Beam Therapy Center (HIT), 69120 Heidelberg, Germany; thomas.tessonnier@med.uni-heidelberg.de; 6 Faculty of Physics and Astronomy, Heidelberg University, 69120 Heidelberg, Germany

**Keywords:** ionizing radiation, FLASH, UNIVERSE, modeling, ultra-high dose rate, temporal pulse structure, electrons

## Abstract

The impact of the exact temporal pulse structure on the potential cell and tissue sparing of ultra-high dose-rate irradiation applied in FLASH studies has gained increasing attention. A previous version of our biophysical mechanistic model (UNIVERSE: UNIfied and VERSatile bio response Engine), based on the oxygen depletion hypothesis, has been extended in this work by considering oxygen-dependent damage fixation dynamics on the sub-milliseconds scale and introducing an explicit implementation of the temporal pulse structure. The model successfully reproduces in vitro experimental data on the fast kinetics of the oxygen effect in irradiated mammalian cells. The implemented changes result in a reduction in the assumed amount of oxygen depletion. Furthermore, its increase towards conventional dose-rates is parameterized based on experimental data from the literature. A recalculation of previous benchmarks shows that the model retains its predictive power, while the assumed amount of depleted oxygen approaches measured values. The updated UNIVERSE could be used to investigate the impact of different combinations of pulse structure parameters (e.g., dose per pulse, pulse frequency, number of pulses, etc.), thereby aiding the optimization of potential clinical application and the development of suitable accelerators.

## 1. Introduction

The biological effects of ultra-high dose-rate (uHDR) ionizing radiation has been investigated since at least the 1960s [1] but has lately been receiving ever-growing attention with numerous reports of the so-called FLASH effect, which is the observation of reduced normal tissue toxicity and preserved tumor control when uHDR are applied at a high dose per fraction [2,3,4]. The underlying mechanism driving the differential sparing between normal tissue and the tumor remains unclear [4,5]. However, while its consistency has been questioned recently [6] and alternative mechanisms (e.g., radical-radical interactions [7]) have been proposed, the radiochemical depletion of oxygen and the resulting transient hypoxia has been one of the first and most discussed mechanisms proposed to explain the general sparing of cells and tissue at uHDR [1,3,4,6,8]. The radioprotective effect of hypoxia is commonly explained by the oxygen fixation hypothesis, whereby molecular oxygen interacts readily with radicals induced in the DNA (damage fixation), preventing their reaction with free H^+^, which would restore their previous state (chemical repair) [9,10]. Based on this mechanism or other radiochemical processes, such as radical recombination, models of biological radiation action have been recently proposed [7,8,11]. However, the validity of their prediction has yet to be extensively tested against experimental data.

In a previously published version of our mechanistic model of radiation action, the “UNIfied and VERSatile bio response Engine” (UNIVERSE) [12], we included the mechanisms of oxygen depletion and re-oxygenation using an analytical description proposed by Petersson et al. [8] and implemented the irradiation process as a quasi-continuous series of damage inductions following the given mean dose rate. The model successfully predicted trends of cell survival as a function of dose, mean dose rate, and environmental oxygen level, while considering the radiation quality, DNA damage repair kinetics and cell-line. However, it has become apparent that the mean dose rate on its own is not a sufficient metric to describe the emergence of sparing effects and the focus has shifted to the concrete description of the applied temporal pulse structure of uHDR radiation, including parameters such as the dose per pulse, number of pulses, delivery time and pulse repetition rate [4,13]. An improved understanding of the interplay between these parameters and their impact on potential sparing effects is crucial for the optimization of possible clinical applications and development of suitable accelerators. Typical pulse-repetition rates applied in uHDR studies are in the order of hundreds of Hz, even exceeding 1 kHz in some cases [4], applying pulses of lengths in the order of microseconds to nanoseconds. Furthermore, Bourhis et al. suggested that the total irradiation time had to be kept below 200 ms to observe the FLASH effect in their review [13]. At these timescales, the kinetics of the damage fixation by molecular oxygen may become relevant. From their experiments in the late 1970s, Watts et al. [14] and Ling et al. [15] concluded that the half-life time of damage fixation was approximately 1 ms [14] and most damages had to be fixed within about 3 ms [15]. 

In this study, we modify our previous implementation of UNIVERSE to consider the concrete temporal pulse structure of the applied radiation and extend it by an oxygen and time-dependent damage fixation mechanism. The model extension is benchmarked using the data obtained by Watts et al. and Ling et al., where an oxygen diffusion function was added to account for setup-specific re-oxygenation dynamics. Furthermore, we consider a reduction in the assumed oxygen depletion rate constant in comparison to our previous implementation and parametrize its tendency to increase at lower dose rates, based on measurements made by Cao et al. [16]. Lastly, we recalculate our predictions for datasets used as benchmarks in our previous publication, to verify that UNIVERSE retains its predictive power in its updated form. Ultimately, this extension of UNIVERSE provides a framework to assess the impact of temporal beam-structure parameters and further investigate the role of oxygen depletion in the observed sparing at uHDR.

## 2. Results

### 2.1. Modeling the Sub-Milliseconds Kinetics of Damage Fixation in UNIVERSE

In order to benchmark the extension of UNIVERSE presented here, we have compared its predictions against in vitro experimental data presented by Watts et al. [14] and Ling et al. [15]. Both publications utilized a setup, in which the cells were not covered in medium during irradiation and thus were directly exposed to the gas mixture within the system. The re-oxygenation of the cells was therefore thought to be driven by diffusion from the gas within the setup into the cells and was considered in our model by using the solution of a one-dimensional diffusion equation (Equations (10) and (11)). 

**Figure 1:**
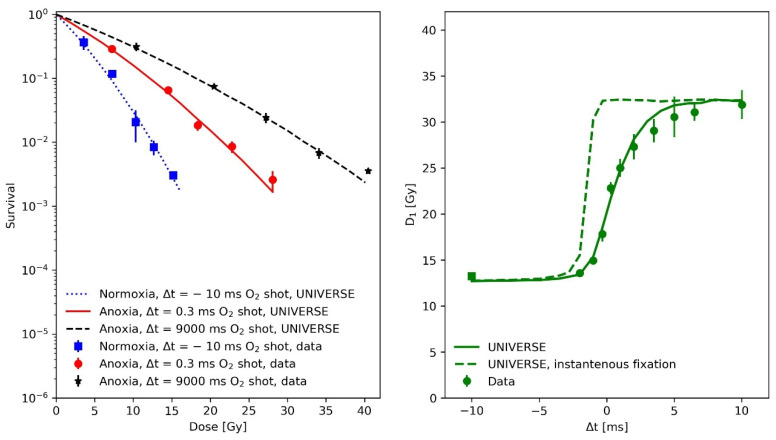
(**Left panel**): Measured (filled circles) and predicted (lines) V79 379A cell survival after a 5 ns electron pulse under various conditions and of O_2_ contact times: squares and dotted line, normoxic condition with O_2_-shot contact 10 ms before electron pulse; stars and dashed line, anoxic condition with O_2_-shot contact 9000 ms after electron pulse; and circles and solid line, anoxic condition with O_2_-shot contact 0.3 ms after electron pulse. (**Right panel**): dose of a 5 ns electron pulse required to achieve a constant 1% survival (D_1_) as a function of O_2_-shot contact time: square, normoxic condition data with O_2_-shot contact 10 ms before electron pulse; and circles, anoxic condition data with O_2_-shot contact at variable time. Positive and negative Δt represent O_2_-shot contact at the times indicate after or before irradiation. Δt = 0 represents simultaneous irradiation and O_2_–shot contact. The solid line depicts model predictions including the oxygen-dependent fixation of damages with a half-life of 1 ms, while the dashed line shows the effect of an assumed instantons damage fixation (half-life = 0 ms). Data taken from [14].

In the first paper, Watts and colleagues used the so-called gas-explosion technique, i.e., combining a fast oxygen gas transfer into the irradiation chamber with a single pulse of radiation, to study the fast kinetics of the oxygen effect in irradiated mammalian cells (Chinese hamster cells V79 379A). Each irradiation consisted of a single 5 ns pulse of electrons of approximately 400 keV mean energy. Based on the data from this experiment, the mean half-life of the damage fixation and the oxygen diffusion parameters were derived for our model extension. In the left panel of Figure 1, survival data (filled circles) and UNIVERSE predictions (lines) are shown for the following three different conditions of O_2_ exposure: squares and dotted line for a normoxic condition with the O_2_-shot contact 10 ms before the electron pulse; stars and dashed line, for an anoxic condition with the O_2_-shot contact 9000 ms after the electron pulse; and circles and solid line, for an anoxic condition with the O_2_-shot contact 0.3 ms after the electron pulse. In the righthand panel of Figure 1, the dose required to achieve a constant 1% survival (D_1_) as a function of O_2_-shot contact time is displayed (circles: experimental data, solid line model prediction). Positive and negative Δt represent O_2_-shot contact times after or before the irradiation, respectively, while Δt = 0 represents simultaneous irradiation and O_2_-shot contact. The UNIVERSE predictions shown in Figure 1 were performed assuming a damage fixation half-life $T_{fix}^{1/2}$ of 1 ms. The dashed line in the right panel of Figure 1 assumes an instantaneous fixation of the damage with a clear overestimation of D_1_. It was found that the data were best described by the model when the oxygen depletion rate constant g was reduced to 70% of the value assumed in our previous publication ($g=0.053\;{\mathrm{Gy}}^{-1}\cdot 0.7=0.037\;{\mathrm{Gy}}^{-1}$). The numerical values of the other model parameters are provided in Table 1. As DNA damage repair does not have any impact on this analysis due to the short timescale, the DNA damage repair parameters were set to representative values.

**Figure 2:**
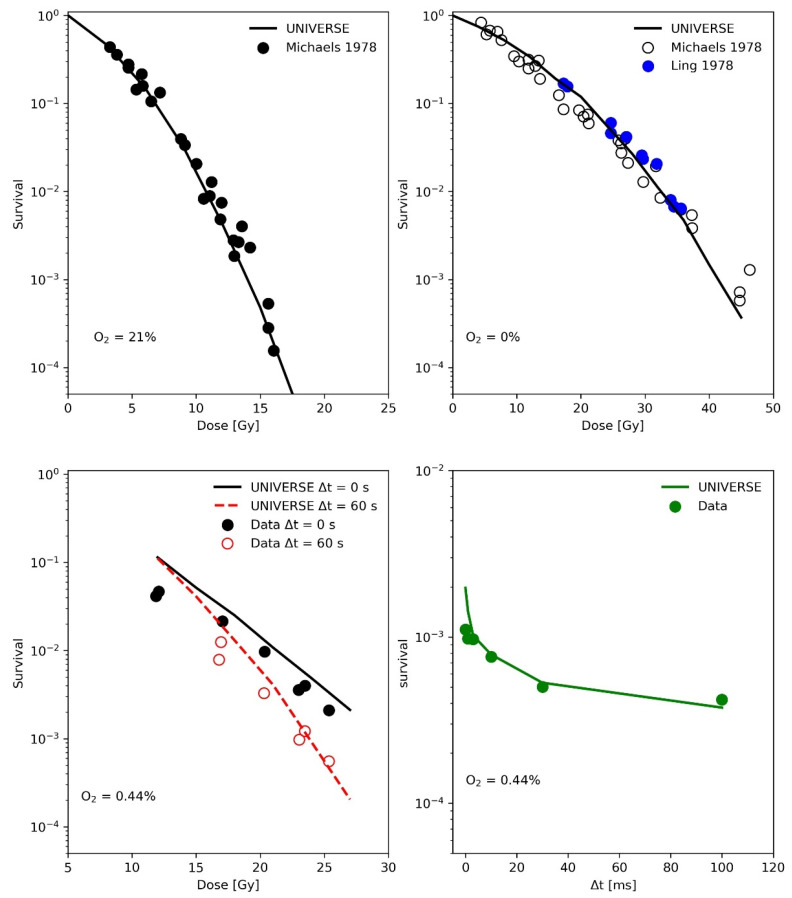
(**Upper-Left panel**): Measured (filled circles) and simulated (line) CHO cell survival as function of dose under normoxic conditions with a 3 ns electron pulse. Data taken from [17]. (**Upper -Right panel**): Measured (filled circles) and predicted (line) CHO cell survival as function of dose under anoxic conditions with a 3 ns electron pulse. Data provided by the same group but presented in different publications [15,17] are shown as reported in the legend. (**Lower-Left panel**): Measured (filled circles) and simulated (lines) surviving fraction of CHO cells irradiated by two 3 ns pulses (first pulse with a fixed dose of 12 Gy, second pulse with variable dose) separated in time by inter-pulse time (Δt) of 0 s or 60 s as reported in the legend. The cells were equilibrated in a 0.44% O_2_ atmosphere in the irradiation chamber. (**Lower-Right panel**): Measured (filled circles) and predicted (line) surviving fraction of CHO cells irradiated with two electron pulses separated by various inter-pulse time Δt. Again, the cells were equilibrated in the irradiation chamber with an atmosphere containing 0.44% O_2_. Data taken from [15].

In the publication by Ling and colleagues [15], two high-intensity electron pulses produced by 600-kV field emission generators, each of 3 ns duration, were delivered to a monolayer of CHO (Chinese hamster ovary) cells in equilibrium with a known concentration of oxygen in the atmosphere of the irradiation chamber. The second pulse was delivered after an accurately identified inter-pulse time, variable from 10^−6^ s to 60 s, in order to study the average lifetime of the radiation-induced oxygen-dependent damage. The upper panels of Figure 2 show the measured (filled circles) and predicted (line) CHO cell survival as a function of single 3 ns electron pulses under normoxic (upper-left panel) and anoxic (upper-right panel) conditions. The measured data were partially obtained by the same group with the same setup but published separately [17] as indicated in the figure legends. These survival curves were used to determine the cell-line specific parameters in UNIVERSE (shown in Table 1), while the same diffusion function and half-life time for the damage fixation, as well as the reduced oxygen depletion rate constant, were used as derived from the data obtained earlier by Watts et al. [14]. In the lower left panel of Figure 2, the measured (filled circles) and predicted (lines) surviving fraction of CHO cells irradiated by two 3 ns pulses (first pulse with a fixed dose of 12 Gy, second pulse with variable dose) separated in time by inter-pulse time (Δt) of 0 s or 60 s as reported in the figure legend are presented. The cells were equilibrated in a 0.44% O_2_ atmosphere in the irradiation chamber. In the lower right panel of Figure 2 the measured (filled circles) and simulated (line) surviving fraction of CHO cells irradiated with two electron pulses separated by various inter-pulse time Δt is shown. Again, the cells were equilibrated in the irradiation chamber with an atmosphere containing 0.44% O_2_.

### 2.2. Resulting O_2_ Depletion in UNIVERSE vs. Experimental Data in the Literature

**Figure 3:**
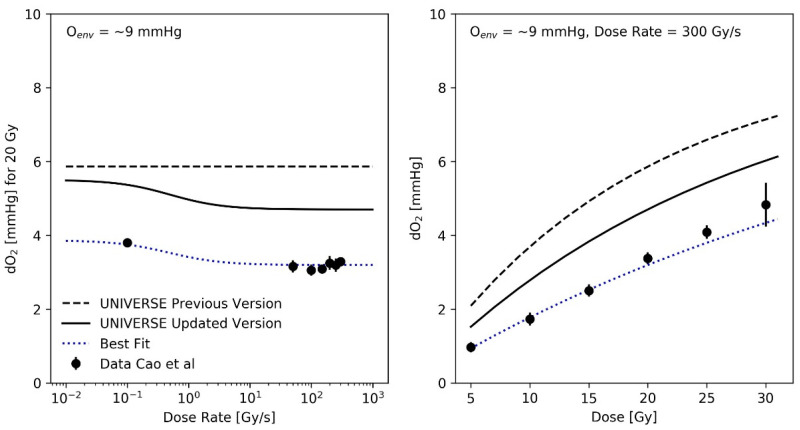
(**Left panel**): Measured oxygen depletion (filled circles) upon 20 Gy irradiation of aqueous solutions (containing bovine serum albumen [BSA] 5% *w*/*w*) as function of dose rate obtained from Cao et al. [16] as compared against calculated values (dashed black line: previous implementation with constant g, dotted blue line: best fit parametrization of g using Equation (12) with Γ=0.022, μ=1.28, κ = 0.45, black solid line: modified parametrization of g using Equation (12) with Γ=0.037, μ=1.28, κ=0.45). The modified parametrization converges to $g=0.037\;{\mathrm{Gy}}^{-1}$ at high dose rates, as implied by prior findings. (**Right panel**): Measured oxygen depletion (filled circles) upon 300 Gy/s irradiation of aqueous solutions ([BSA] 5% *w*/*w*) as a function of dose, obtained from Cao et al. [16] and predictions resulting from each parametrization.

The preceding analysis implied a reduction of the oxygen depletion rate constant g by a factor of 0.7 in comparison to our previous implementation [12], resulting in a value of $g=0.037\;{\mathrm{Gy}}^{-1}$. As an exemplary comparison, the oxygen depletion measurements performed by Cao et al. [16] using a standard phosphate-buffered saline solution containing 5% Bovine serum albumin (BSA) to mimic the presence of biological molecules in sealed glass vials are plotted as a function of the dose rate and as a function of dose (for a fixed dose-rate of 300 Gy/s) in the right and left panel of Figure 3, respectively, for an environmental atmospheric oxygen level (O_env_) of approximately 9 mmHg. It is important to note that the calibration of the method employed by Cao et al. results in the reading of the oxygen concentration to correspond to the concentration in the environmental atmosphere, with which the solution would be in an (gas-exchange) equilibrium with [18]. In principle, this facilitates a direct comparison of their results with our description of the oxygen dynamics, which is based on a parametrization of the oxygen effect using the atmospheric oxygen concentration as the input. The implied oxygen depletion detected in the previous model implementation (dashed lines in Figure 3) was based on a dose-rate independent oxygen depletion rate constant $g=0.053\;{\mathrm{Gy}}^{-1}$, as suggested by Petersson et al. [8]. Cao et al. (and others [19]) observed an increased amount of oxygen depleted at SDR (for Cao et al. at about 0.1 Gy/s) in comparison to uHDR (Figure 3—left panel). To account for this effect, we used an empirical parametrization of g over the applied dose rate (Equation (12)), using the three phenomenological parameters Γ, μ and κ. As these parameters solely serve to describe the general radio-chemical oxygen depletion mechanism at a given dose rate, they are thought to be mechanistically independent of the parameters in Table 1, which describe a cell line’s intrinsic sensitivity to certain damage classes and its ability to repair them, as well as its sensitivity to a change in oxygen status. The best fit of the experimental depletion data over the applied dose rate (Figure 3, left panel) using the model parameters Γ=0.022, μ=1.28 and κ=0.45, as well as the resulting dose dependency (Figure 3, right panel) are shown as blue dotted lines. A modified parametrization, which conserves the trend over the dose-rate but converges to $g=0.037\;{\mathrm{Gy}}^{-1}$ for high dose-rates, in accordance with prior findings, by setting Γ=0.037 is shown as black solid lines in Figure 3. 

### 2.3. Recalculation of Previous Benchmarks

Cell-survival data from Epp et al. [20] and Adrian et al. [21] were used as benchmarks in our previous publication. The predictions for these setups were expected to be subject to change once the extensions to UNIVERSE presented here were applied, based on the environmental oxygen level applied in their experiments and the resulting sensitivity to a potential oxygen-depletion effect. To ensure that the new version of the model still reproduced the measurements well, their predictions were recalculated using the updated version of the UNIVERSE.

**Figure 4:**
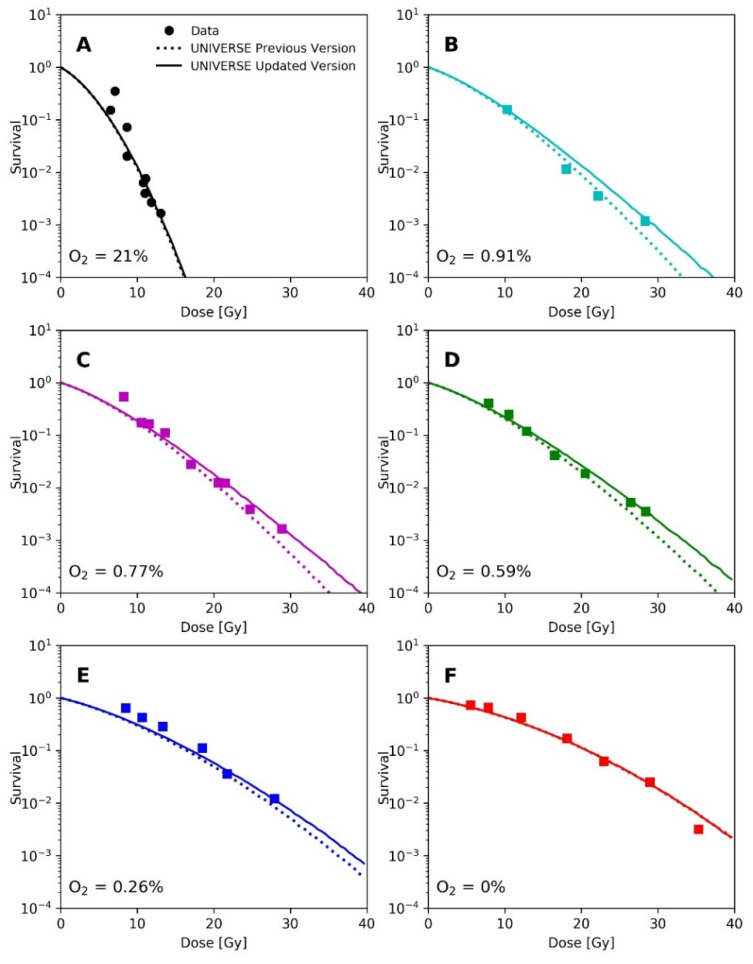
Panels (**A**–**F**): Measured survival (filled circles and squares) of HeLa cells after irradiation with a 3 ns electron pulse under different environmental oxygen levels taken from Epp et al. [20] and respective predictions by previous version of UNIVERSE (dotted lines) and the updated UNIVERSE including the extensions presented in this study (solid lines).

Epp et al. irradiated HeLa cells with 3 ns pulses of electrons with a mean energy of about 350 keV under different environmental atmospheric oxygen levels. Analogous to the methodology applied by Watts et al. and Ling et al., Epp et al. had irradiated the cell monolayer after removing the medium [14,15,20]. Thus, we applied the same diffusion function utilized earlier for the prediction of the data from Watts et al. and Ling et al. for this analysis. The depletion-rate constant was also set to $g=0.037\;{\mathrm{Gy}}^{-1}$. The cell-line dependent UNIVERSE parameters were adopted from our previous publication [12] and are shown in Table 1. In the panels of Figure 4, their measured survival curves (filled circles and squares) are shown for the different environmental oxygen levels, together with the predictions of our previous model (dotted lines) and the new extension (solid lines). 

Adrian et al. [21] measured the survival of DU145 cells after irradiation with 10 MeV electrons at a conventional dose-rate (0.23 Gy/s) and uHDR (600 Gy/s) under normoxic and hypoxic (1.6% O_2_) atmosphere. The uHDR was realized by applying pulses of 3 Gy at a rate of 200 Hz. For our predictions, we applied the same pulse repetition rate (200 Hz) for the conventional dose-rate, scaling the dose per pulse to 1.15 mGy to match the reported mean dose-rate of 0.23 Gy/s. As the cells were in the medium during irradiation, the re-oxygenation process was described using the differential equations proposed by Petersson et al. [8] (Equations (4) and (5)), which had been part of our previous implementation. The cell-line dependent UNIVERSE parameters were adopted from our previous publication [12] and are shown in Table 1.

## 3. Discussion

The analysis of the measurements by Watts et al. [14] (Figure 1) have shown that the implementation of a fixation half-life time $T_{fix}^{1/2}$ is necessary for UNIVERSE to describe kinetics in the millisecond range which affect cell survival. Assuming that the instantaneous fixation of the damage (dashed line, right panel Figure 1) prevents the damages from coming into contact with the oxygen provided by any O_2_ shot delivered after the irradiation, leading to an instantaneous jump of the predicted D_1_ at Δt = 0 and a clear overestimation of the value in comparison to the data as a result. However, the application of a $T_{fix}^{1/2}$ of 1 ms, as approximated by Watts et al. [14] and in accordance with the later findings of Ling et al. [15], allows UNIVERSE to match the measured trend well. As their experimental procedure included removing the medium from the cells shortly before irradiation, the setup-specific re-oxygenation kinetic for the measurements by Watts et al., Ling et al. and Epp et al. [14,15,20], were described using the solution of a one-dimensional diffusion equation (Equations (10) and (11)), as proposed by Ling et al. [15]. The latter group irradiated CHO cells with electron pulses in quick succession to characterize the diffusion process. We used their data to further benchmark our implementation (Figure 2) and were able to successfully predict the observed trends. However, we found that a cell-line independent effective diffusion distance of 7 µm was able to match the data, while Ling et al. found a shorter value of 4 µm. This discrepancy could be traced back to the fact that Ling et al. equated the fractional oxygen that had rediffused into the system to the relative shift in the logarithm of survival [15], while, in UNIVERSE, the rediffused oxygen would be considered as an input to our non-linear parametrization of DNA double-strand breaks (*DSB*) yield reduction (Equation (6)), which then serves as a measure for the probability of a *DSB* becoming fixated at a given timepoint. While the values for the diffusion distance are comparable to typical cell radii [15,22], it is of importance that the general application of the diffusion equation poses a significant simplification of the system, ignoring cell morphology, membrane permeability and diffusion through different chemical milieus, in which further reactions could occur. The diffusion distance thus represents an “effective” distance, assuming pure water as a medium. As Ling et al. [15] indicated in their work, calculations could in principle consider such details; however, too many tenuous assumption would have to be included and their study, as well as our analysis, have shown that the applied model is sufficient to capture the observed trends to more than a reasonable degree, especially considering its use as a purely auxiliary model. If found necessary, cell-line dependencies could be introduced in the future via the effective diffusion distance.

**Figure 5:**
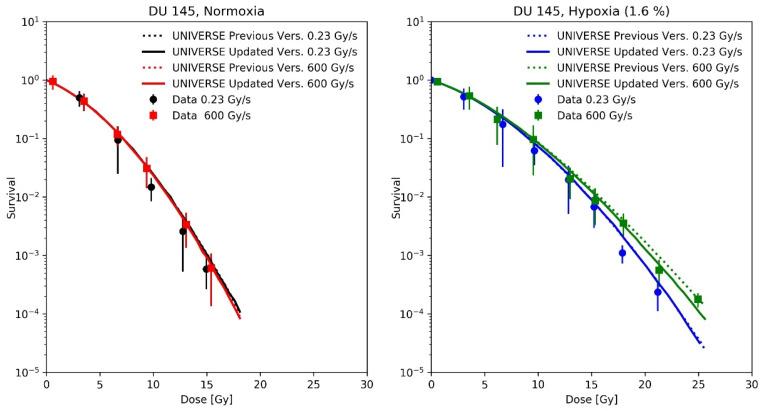
Measured cell survival of DU145 cells taken from Adrian et al. [21] after irradiation with 10 MeV electrons at conventional dose-rate (circles, 0.23 Gy/s) and uHDR (squares, 3 Gy per pulse and 200 Hz = 600 Gy/s) under normoxic, (**Left panel**) and hypoxic (1.6% O_2_, **Right panel**) atmosphere with predictions based on previous implementation of UNIVERSE (dotted lines) and updated UNIVERSE including the extensions presented in this study (solid lines).

A recalculation of our predictions for the measurements of Epp et al., using the proposed extension (Figure 4), leads to an increased predicted sparing effect and general improvements in the predictions for the hypoxic cases (Figure 4D,E), while for the normoxic (Figure 4A) or anoxic case (Figure 4F) sparing remains absent due to the large amount or absence of available oxygen, respectively [12]. However, the prediction in Figure 4B appears to have worsened slightly. The underestimation of measured survival at lower doses exhibited by both versions of the model could be in part explained by the lack of correction for cellular multiplicity during the clonogenic assays by the authors [20,23]. The increase in predicted sparing despite a reduction in the oxygen-depletion rate constant can be explained as follows. In the previous version of UNIVERSE, where the damage fixation was assumed to be instantaneous, the dose applied by the pulse was divided into small consecutively applied fractions in which the induced damages were only affected by the oxygen depletion caused by the current and previously applied fractions. In the presented extension, however, the 3 ns pulse applied by Epp et al. was significantly shorter than $T_{fix}^{1/2}$, so that virtually all induced damages are affected by the entire oxygen depletion caused by the electron pulse, reducing the overall number of induced damages. The dose was not applied in a single pulse in the setup used by Adrian et al. [21] (Figure 5); instead, the dose was “bunched” into 3 Gy pulses for the uHDR setting, decreasing the average amount of oxygen available to the induced damages in comparison to the former implementation. This effect appears to at least partly compensate for the reduction in the oxygen-depletion rate constant, so that the updated version of UNIVERSE is required to provide an accurate prediction of the measured cell survival.

A fixation mechanism of damages on the timescale of milliseconds leads to several interesting implications. First, any pulse of radiation that is significantly shorter than the fixation half-life time would be virtually instantaneous for the system. In other words, as soon as the pulse length is far shorter than the fixation half-life time, the intra-pulse dose-rate becomes irrelevant. This presupposes, that the pulse length is also much shorter than the timescale of the given re-oxygenation kinetics. Second, the sparing effect could be enhanced by increasing the amount of oxygen depletion before a significant amount of damage is fixated, as seen for the predictions of the data taken from Epp et al. and Adrian et al. This could be achieved by two, non-mutually exclusive approaches. i.e., increasing the dose per pulse and/or increasing the pulse repetition rate to a level at which additional depletion can occur within the fixation process of a previous pulse. The latter effect would be expected at pulse repetition rates of $⪆1/T_{fix}^{1/2}$, which for the assumed fixation half-life time of 1 ms in this study would be equal to ⪆1 kHz. In our previous publication [12], we argued against the statement of a specific threshold values of certain parameters (i.e., dose and dose-rate) that would lead to the observation of a sparing effect due to the multitude of relevant variables. Similarly, one should probably not attempt to impose rigid thresholds for the dose-per-pulse or pulse repetition rate, but rather assess their interplay and its effect based on a given range of options (i.e., provided by the setup and its technical capabilities). We believe that the extension of UNIVERSE presented here could serve as a basis for such an analysis, supporting the development of novel accelerators and an understanding of the experimental results.

Recent experimental studies found a tendency of higher oxygen depletion per applied unit dose at lower dose rates in comparison to uHDR [16,19]. To consider this effect, an empirical parametrization was found that could describe the trends over the applied dose and dose rate observed in the dataset measured by Cao et al. (Figure 3). The recalculation of the predictions for the measurements of Adrian et al. (Figure 5) did continue to show an observable sparing effect and matched the data well, despite the introduction of different oxygen-depletion rate constants for the conventional and uHDR irradiation settings. In our earlier implementation, the oxygen-depletion rate constant *g*, used in the description of the oxygen-depletion mechanism was adopted as a constant value from the work by Petersson et al. [8] (dashed line in Figure 3). While the presented analysis implied a reduction in the oxygen-depletion rate constant to about 70% of the value used (solid line) in our previous implementation, the resulting value necessary to explain the observations remains higher than those measured by Cao et al. (filled circles). For the setting presented in the right panel of Figure 3 (environmental oxygen ~9 mmHg, dose-rate = 300 Gy/s), the oxygen depletion in our model after 10 Gy was about 1 mmHg (~0.13% oxygen) above the measured value. Two main reasons for this discrepancy are considered: 

First, although Cao et al. tried to consider the presence of biological molecules by adding BSA to the irradiated vials, they commented that the amount of oxygen depletion is not only dependent on the concentration but also the exact composition of compounds present in the irradiated solution [16], lending a possible explanation for the deviation between their measurements and the values required in UNIVERSE to describe the sparing, as the chosen medium might not correctly replicate the chemical environment in the cell nucleus. Spitz et al. [24] even supposed that due to lipid-peroxidation chain reactions and redox-active iron, the oxygen depletion in certain cells might be up to four times as high as the value in water or a cell-culture medium [11]. However, in a recent article, Wardman [25] pointed out that several recent studies of radiation chemistry at uHDR, including that of Spitz et al., included oxygen depletion by species which would be strongly suppressed in cells due to the high concentrations of competing scavengers. Furthermore, he pointed out that lipid peroxidation is a relatively slow process, for which the timescale might significantly exceed the time considered for damage fixation and thus might only play a role for repeated pulse exposures. 

Second, open questions also appear with regard to possible radical–radical interaction mechanisms [25], a potential alternative explanation for the sparing effect at uHDR proposed already by Berry [26] in the late 1960s and recently implemented by Labarbe et al. [7]. The disregard of such a competing mechanism could be another possible explanation for the deviations between the measured amount of oxygen depletion by Cao et al. and the values necessary to explain sparing effects in UNIVERSE. Yet, it remains challenging to theoretically and experimentally characterize the relevant processes within the cell’s nucleus that contribute to the sparing effect.

As mentioned in our previous publication [12], UNIVERSE is not bound to the oxygen depletion hypothesis per se and could in principle be driven by any other mechanism that ultimately affects the number of *DSB*. However, in this study, we were able to show that with the presented inclusion of oxygen-dependent damage fixation kinetics, UNIVERSE is able to explain the observed sparing kinetics in the timescale of milliseconds and retain its predictive power even after the inclusion of a dose-rate-dependent and reduced oxygen-depletion rate constant approaching the measured values. 

## 4. Conclusions

The UNIVERSE framework was extended using an explicit description of temporal-beam structures including an oxygen-dependent damage fixation kinetic, which was found to be necessary to explain the sub-millisecond dynamics of in vitro survival data. The modified model was shown to retain or improve its predictive capabilities in benchmarks already applied to the previous implementation of the software, while the assumed amount of oxygen depletion could be lowered in comparison to the earlier version to approach measured values, even when considering an increase in the depletion rate with a decreasing applied dose rate, as implied by recent reports. However, the assumed amount of oxygen depletion remains above the measurements currently available in the literature. Further investigation is needed to understand whether this discrepancy can be explained by the incomplete replication of the chemical environment within the cell nucleus in the published oxygen-depletion measurements, or if further mechanisms need to be implemented into our model. The findings of this study may provide valuable contributions to the ongoing debate, of whether oxygen depletion is a viable mechanism by which to explain at least some of the sparing at uHDR. In practice, the updated UNIVERSE could be useful to investigate the impact of different combinations of pulse-structure parameters (e.g., dose per pulse, pulse frequency, number of pulses, etc.), which has gained increasing attention in the field, as well as in aiding the optimization of potential clinical application and the development of suitable accelerators. 

## 5. Materials and Methods

### 5.1. Experimental Data from Literature

The experimental survival data used to benchmark the extensions of our model were taken from [14,15,16,17,20,21]. 

### 5.2. Modeling Approach

UNIVERSE is a mechanistic model of radiation action in biological systems which has been used to describe low LET (linear energy transfer) and higher LET radiation both alone and in combination with genetic or pharmaceutical DNA damage repair interference [27,28]. Furthermore, a mechanism to model the effect of radical scavengers for low LET radiation was implemented [29]. In order to predict the effect of various dose rates, ranging from the lowest clinical settings up to the uHDR range, the low LET version of UNIVERSE was extended to consider DNA-damage repair, oxygen depletion and re-oxygenation kinetics [12]. 

In general, sparsely ionizing radiation (e.g., X-rays, gamma rays, electrons) is thought to deposit the dose homogeneously throughout the target. The total number of DNA double strand breaks (*DSB*) expected to be induced in a cell nucleus ($\langle N_{tDSB}\rangle$) is then assumed to be given by:

(1) $$\langle N_{tDSB}\rangle =\alpha_{DSB}\cdot D,$$

where *D* is the applied dose and $\alpha_{DSB}$ is the average yield of *DSB* per cell per unit dose. This yield is assumed to be $\alpha_{DSB}=30\frac{DSB}{Gy\cdot Cell}$ [30] and to be constant over the clinical dose range [31]. In UNIVERSE, the chromatin is subdivided into so-called giant loops, which contain about 2 Mbp of DNA [32,33,34,35,36] that are classified as *isolated DSB* (*iDSB*) when they contain exactly one *DSB* and as complex or *clustered DSB* (*cDSB*) when they contain two or more *DSB*. This classification is shared with other models [37,38] and was successfully used to predict the proportions of swiftly and slowly repairing damages in the data of the rejoining studies [39,40]. To simulate the survival fraction of a cell population, the total number of *DSB* was sampled from a Poisson distribution with the expectation value $\langle N_{tDSB}\rangle$ for $>10^{4}$ iterations. The sampled *DSBs* were randomly assigned to the domains within the nucleus and the number of *isolated DSB* ($N_{iDSB}$) and *complex DSB* ($N_{cDSB}$) were scored. Given theprobabiliy of one *isolated DSB* or one *complex DSB* inactivating the cell as $K_{iDSB}$ and $K_{cDSB}$, respectively, the overall probability of a cell surviving irradiation (*S*) can be written as [27,38]:

(2) $$S={\left(1-K_{iDSB}\right)}^{N_{iDSB}}\cdot {\left(1-K_{cDSB}\right)}^{N_{cDSB}}$$

The parameters $K_{iDSB}$ and $K_{cDSB}$ are cell-line dependent fit-parameters and the average value of *S* over all iterations is the predicted survival fraction of the cell population. The values of the so-called lethality parameters, $K_{iDSB}$ and $K_{cDSB}$, are obtained by fitting the data after uHDR irradiation under normoxia. While repair effects are in practice suppressed at uHDR, within our framework [12] (also cp. below) the amount of oxygen depleted in normoxic scenarios by doses within the range usually applied in relevant studies is not sufficient to impact our predictions, allowing for the neglection of possible sparing effects and a direct determination of the lethality parameters. 

In the previous implementation of the dose-rate effect in UNIVERSE [12], the dose was assumed to be applied in a quasi-continuous fashion with a fixed mean dose rate ($\dot{D}$). The total irradiation time ($T_{rad}$) was determined using $T_{rad}=\frac{D}{\dot{D}}$ and was subdivided into a given number of timesteps ($N_{t}$ = 100). At each timestep, the partial dose $D_{part}=\frac{D}{N_{t}}$ was applied to the nucleus. To account for potential oxygen depletion and re-oxygenation during the irradiation, the oxygen level at a given timestep O(t) was computed using [8]:

(3) $$O\left(t\right)=O_{env}\left(\frac{\lambda }{g\dot{D}+\lambda }+\left(1-\frac{\lambda }{g\dot{D}+\lambda }\right)e^{-\left(g\dot{D}+\lambda \right)t}\right)$$

where t is the time elapsed since the start of the irradiation, $O_{env}$ is the environmental oxygen level (pO_2_) at t=0, g is the depletion rate constant and λ is the re-oxygenation constant. Equation (3) is the analytic solution, provided by Petersson et al. [8], to the sum of their description of the oxygen-depletion process

(4) $$\frac{dO}{dt}=-g\dot{D}O\left(t\right)$$

and re-oxygenation process:

(5) $$\frac{dO}{dt}=\lambda \;(O_{env}-O\left(t\right)$$

In our previous publication, we used the depletion rate constant $g=0.053\;{\mathrm{Gy}}^{-1}$ and re-oxygenation rate constant $\lambda =1\;s^{-1}$, as suggested by Petersson et al. [8] in their in vitro analysis. The time-dependent oxygen concentration O(t) was then employed to determine the reduction of damage yield via the hypoxia-reduction factor $HRF_{DSB}^{O_{2}}\left(t\right)$, which can be parametrized as:

(6) $$HRF_{DSB}^{O_{2}}\left(t\right)=\frac{m\cdot K+\left[O\left(t\right)\right]}{K+\left[O\left(t\right)\right]}$$

This parametrization was first proposed by Carlson et al. [41], who were inspired by the original works of Alper and Howard-Flanders [42]. The parameters *m* and *K* are the maximum value and the turning point (in a plot with an x-axis in log-scale) of the described dependency, respectively. Ultimately, the reduced *DSB* yield, $\alpha_{DSB}^{O_{2}}$, is given by:

(7) $$\alpha_{DSB}^{O_{2}}=\frac{\alpha_{DSB}}{HRF_{DSB}^{O_{2}}}$$

If both hypoxic and normoxic data are available, $HRF_{DSB}^{O_{2}}$ can be determined by fitting the model to both datasets, while $K_{iDSB}$ and $K_{cDSB}$ remain constant [12,27,29]. The values of the parameters *m* and *K* were initially considered to be cell-line independent and earlier publications attempted to implement global parametrizations [12,27]. However, there has always been a considerable amount of variation in the best-fit parameters for a number of data sets [12,27], so that in some analyses, including this study, deviating values are used to consider the variability that could arise between cell lines and experimental conditions. 

At this point, it is important to note that the parametrization of the HRF applied in this framework was mainly established and benchmarked based on in vitro data, for which the reported oxygen level exclusively corresponded to the oxygen concentration in the atmosphere in which the cells were cultured and/or irradiated in [27,31,43]. Thus, the time-dependent oxygen level in our description that is ultimately used to describe the change in the damage yield, corresponds to the concentration in the atmosphere in which the cells would have to be irradiated (in equilibrium) to observe the predicted effects.

To account for the repair kinetics of the induced damages, *iDSB* and *cDSB* are allotted a random lifetime sampled from an exponential distribution following the cell-line dependent repair half-life times $T_{iDSB}^{1/2}$ and $T_{cDSB}^{1/2}$, respectively. With $T_{iDSB}^{1/2}$ usually in the order of minutes and $T_{cDSB}^{1/2}$ in the order of hours, repair kinetics do not contribute significantly to biological effects atuHDR. Thus, we kindly refer readers to our previous publication for a more detailed account of our implementation of this process [12].

For this study, the kinetics of oxygen-mediated fixation of damages was implemented into UNIVERSE. The half-life time of the fixation kinetic $T_{fix}^{1/2}$ was assumed to be 1 ms, following the findings by Watts et al. and Ling et al. [14,15]. The fixation process was simulated by sampling a fixation time from an exponential distribution based on $T_{fix}^{1/2}$ for each induced *DSB* followed by checking every $t_{c}$, whether the given fixation time of any damage had elapsed. If the sampled-fixation time for a damage was reached, it was added to the corresponding domain with the probability $1/HRF_{DSB}^{O_{2}}$. While a short $t_{c}$ is computationally expensive, it needs to be short enough to appropriately sample the oxygen kinetics of a given setup. For the setups of Watts et al., Ling et al. and Epp et al. [14,15,20] and the uHDR setting of Adrian et al. [21], a $t_{c}$ of 0.1 ms was chosen, due to their fast oxygen kinetics, while for the SDR setup of Adrian et al. [21] a $t_{c}$ of 5 ms was found to be sufficient. Furthermore, in this extension of the model, O(t) is no longer determined using Equation (3) and a quasi-continuous approach, but is instead based on an explicit implementation of the temporal-pulse structure. After implementing a pulse with a given dose $D_{pulse}$ the oxygen level is given by the solution of Equation (4):

(8) $$O\left(D_{pulse}\right)=O_{pre}\cdot e^{-gD_{pulse}}$$

where $O_{pre}$ is the oxygen level before the pulse was applied. The re-oxygenation between pulses is then determined using the solution of Equation (5):

(9) $$O\left(t_{post}\right)=O_{post}\cdot e^{-\lambda t_{post}}+O_{env}\left(1-e^{-\lambda t_{post}}\right)$$

with $O_{post}$ being the oxygen level after the last pulse and $t_{post}$ being the time that has elapsed since the last pulse. While Equation (9) was applied to the analysis of the data from Petersson et al., a setup-specific function had to be used for the data of Watts et al., Ling et al. and Epp et al. We adopted the approach proposed by Ling et al. [15], which supposed the thin cell layer in their setup to be a semi-infinite medium where the time- and position-dependent oxygen concentration O(x,t) could be described be the one-dimensional diffusion equation:

(10) $$D_{O_{2}}\frac{\partial O\left(x,t\right)}{\partial x^{2}}=\frac{\partial O\left(x,t\right)}{\partial t}$$

where *x* is the depth into the medium and $D_{O_{2}}$ is the diffusion coefficient for oxygen within the medium. The solution is then given by the complementary error function (erfc):

(11) $$O\left(x,t\right)=O_{env}\cdot \frac{2}{\sqrt{\pi }}\;\int_{a}^{\infty }\exp(-\eta^{2})d\eta =O_{env}\cdot \mathrm{erfc}(a)$$

with $a=x/2\sqrt{D_{O_{2}}t}$ and a dummy variable η [15], $D_{O_{2}}$ was set to the value for oxygen in water ($2\cdot 10^{-5}\;{\mathrm{cm}}^{2}s^{-1}$), while the best description of the data was achieved with a diffusion distance of x=7 µm. While this description continues to correspond to the atmospheric oxygen concentration in which the cells would have to be irradiated (in equilibrium) in a standard culturing setup, the consistency of the model is ensured by the standard procedure of calibrating the HRF parameters, as described earlier, to the survival curves with reported atmospheric conditions. By this, it is solely assumed that the functional dependency between the oxygenation level and the yield of damages is of the same general form (Equation (6)) in these setups and in standard culture conditions. 

To describe the trend of *g* over the applied dose rate, we chose the following empirical parametrization:

(12) $$g\left(\dot{D}\right)=\Gamma \cdot \frac{\mu \cdot \kappa +\dot{D}}{\kappa +\dot{D}}$$

The function was chosen based on the assumption that the depletion-rate constant should approach finite values both at very high dose rates (g=Γ for $\dot{D}\to \infty$) and very low dose rates (g=Γ⋅μ for $\dot{D}\to 0$). The chosen parametrization offers a simple sigmoidal form to connect both regimes when the dose rate is shown in a logarithmic scale and was inspired by the function used to describe the oxygen dependency of $HRF_{DSB}^{O_{2}}$ (cp. Equation (6)).

## Figures and Tables

**Table 1:** Cell line dependent UNIVERSE parameters applied in this work. Values marked with a * have been adapted without modification from our previous publication [12].

Cell Line	$K_{iDSB}$	$K_{cDSB}$	$T_{iDSB}^{1/2}\;[\min]$	$T_{cDSB}^{1/2}\;[\min]$	m	K	Reference
V79 379A	9.1 × 10^−3^	0.08	60	300	2.6	0.129	Watts et al. 1978
CHO	5.9 × 10^−3^ *	0.19 *	80.22 *	300 *	3.1 *	0.2	Ling et al. 1978
HeLa	6.7 × 10^−3^ *	0.21 *	14 *	130 *	3.4 *	0.41 *	Epp et al. 1972
DU145	5.9 × 10^−3^ *	0.17 *	4 *	100 *	3.1 *	0.27 *	Adrian et al. 2019

## Abbreviations

LET	Linear energy transfer
SDR	Standard dose rate
uHDR	Ultra high dose rate
UNIVERSE	UNIfied and VERSatile bio response Engine
DSB	(DNA) Double strand break
iDSB	Isolated DSB
cDSB	Complex DSB
OER	Oxygen enhancement ratio
HRF	Hypoxia reduction factor
CHO	Chinese hamster ovary
